# Bridging the gap between policy and practice: a mixed-methods study of tobacco control implementation challenges and community responses in Hyderabad, Sindh, Pakistan

**DOI:** 10.3389/fpubh.2026.1881029

**Published:** 2026-06-18

**Authors:** Juanmiao Shi, Gu Jintu, Abdul Rasool Khoso, Saeed Ahmad

**Affiliations:** 1 Department of Sociology, School of Public Administration, Hohai University, Nanjing, China; 2 School of Law and Social Work, Zhejiang University of Finance and Economics, Dongfang College, Jiaxing, China

**Keywords:** cessation services, CFIR, health policy, implementation science, LMIC, mixed-methods, Pakistan, tobacco Control

## Abstract

**Background:**

Pakistan is a party to the WHO Framework Convention on Tobacco Control and has enacted comprehensive tobacco control legislation, yet implementation remains weak. A mixed-methods study was conducted to explore implementation challenges, community response, and the health impact of this initiative among adult males in Hyderabad, Sindh.

**Methods:**

A convergent parallel design included 600 male adults (18–60 years) selected through multistage stratified random sampling and 15 in-depth interviews. Quantitative data were analyzed using structural equation modeling (SEM); qualitative data were analyzed thematically, guided by the Consolidated Framework for Implementation Research (CFIR).

**Results:**

The prevalence of tobacco use was 67% (402/600). Among users, 52% had attempted to quit, while 0% had accessed cessation services despite wanting to quit. Low policy awareness: 16% were aware of cessation services, and 46% were aware of the smoke-free law. Most believed minors could easily obtain tobacco (74% thought enforcement was weak). The health burden also tended to be high: 76% of users showed tobacco-related symptoms, compared with only 43% of non-users (*p* < 0.001). The model fit the data well (CFI = 0.94, RMSEA = 0.048). Weak policy implementation directly predicted higher tobacco use (*β* = −0.41, *p* < 0.001), which in turn predicted poorer self-reported general health (*β* = 0.53, *p* < 0.001). Qualitative themes identified enforcement failures, the cultural normalization of noncompliance with public health laws, constructs within the healthcare system that enabled noncompliance, and economic constraints on enforcement.

**Conclusion:**

The male population in Hyderabad faces a significant implementation gap, with the most severe failure being the absence of cessation services. Limitations, including male-only sampling, self-reported data, and a single-district focus, prevent generalization to women and to the whole of Pakistan. Immediate policy action is required to establish accessible cessation support, strengthen enforcement mechanisms, and integrate tobacco control into routine healthcare.

## 1 Introduction

Tobacco use is the leading preventable cause of morbidity and mortality worldwide, responsible for >8 million deaths each year (1, 2), and it also accounts for significant health care costs as well as economic losses at the national level (3). Despite decades of worldwide initiatives to curb tobacco use, this commodity continues to rank among the most significant threats to global public health (4). Over 80% of the 1.3 billion tobacco users live in low- and middle-income countries (LMICs), where almost nine-tenths of the global burden of tobacco-related ill-health and death falls (5). The World Health Organization Framework Convention on Tobacco Control (FCTC) which was approved in 2002 and has been ratified by 182 countries including Pakistan (6), contains a comprehensive evidence-based package for tobacco control which includes the MPOWER measures: M: monitor tobacco use; P: protect people from second-hand smoke; O: offer help to quit; W: warn about the dangers of tobacco; E: enforce advertising bans; R: raise taxes on tobacco products (7, 8). Such measures, including large graphic health warnings adopted by 103 countries that protect nearly 4.5 billion people and full smoke-free laws covering over one-quarter of the world’s population, have shown great effectiveness globally (9).

The implementation of tobacco control policies across regions and countries is driven by the political, economic, and sociocultural interests of actors from various sectors (10). Cross-country data from the WHO European Region indicate substantial variation in FCTC and MPOWER implementation, with the proportion of required measures fully operationalized ranging from 28 to 86%. Notably, nations that have formally adopted tobacco endgame targets consistently report markedly higher composite implementation scores compared to those without such goals (11). The UK, through the proposed Tobacco and Vapes Bill 2024, has introduced a new initiative, the Tobacco-Free Generation Initiative, whose goal is to prevent people born on or after January 1, 2009, from ever legally buying tobacco products (12). Even Nordic countries have modern policies such as point-of-sale display bans in Iceland (13), outdoor smoking bans in Sweden (14), flavor bans for e-cigarettes in Finland and plain packaging in Texas, Norway and Denmark (15, 16). In the Eastern Mediterranean region, 13 out of 22 countries apply taxes greater than 50% retail prices, health warnings are mandatory nearly everywhere in the region, and five nations, Bahrain, Jordan, Kuwait, Qatar, and Saudi Arabia, entirely pay for quitting treatment (17). India, located within the WHO South-East Asia Region, has enacted the Cigarettes and Other Tobacco Products Act (COTPA) of 2003, which prohibits tobacco sales to minors and within a 100-yard radius of educational institutions. Additionally, the Prohibition of Electronic Cigarettes Act of 2019 forbids the manufacturing, production, importation, and distribution of e-cigarettes across the country (18). A community-based case study in rural Rajasthan showed that multi-stakeholder engagement at the local level enhanced tobacco control, such as improved awareness among people (as measured by the improved Tobacco-Free Educational Institution scores → Score Baseline Pre-Intervention = 9 Post intervention = 90) (19). Australia led plain packaging for cigarettes in the Western Pacific Region (2012) (20), and China’s Healthy China 2030 strategy aims to achieve a smoking rate of 20% through development of legislation on smoke-free public places, including ban on tobacco use in educational institutions (21). Yet, the African region continues to make slow progress with only a few countries complying with FCTC requirements in full (22).

Using the Index of Tobacco Control Sustainability (ITC S) to measure progress in tobacco control, four Asian countries were assessed from 2016 to 2022, and significant gains in score were seen in two of them, with Myanmar making advances from 43 to 82 and Indonesia from 54 to 74 over 6 years (23). These advances reflect strengthened policy implementation, structural capacity, and mobilization of resources through new mechanisms of Indonesia’s subnational tax allocations (24). However, considerable obstacles remain in terms of developing capacity, sponsoring research and instituting effective tobacco taxation policies especially in resource-limited environments (25). The case of Afghanistan illustrates how implementation deficits can persist even within resource-constrained health systems. Although the country has ratified the FCTC, enforcement of national smoke-free legislation remains consistently weak, no national telephone-based quit support service has been established, and evidence-based cessation pharmacotherapy is unobtainable through legal channels (26), health warnings limited to text-only format and implemented inconsistently, while access to cigarettes has become relatively more affordable since 2008 because of very low levels of taxation (27). Tobacco, the leading preventable cause of death in Afghanistan, kills more than 15,000 Afghans each year, contributing to over 12% of all male deaths and yet tobacco companies freely advertise through retail and informal markets without online buying (28). The large gap between the high rates of policy adoption against far more modest rates of effective implementation across low-and middle-income countries emphasizes the urgent need for local level studies investigating what is feasible to implement.

### 1.1 Statement of problem

Pakistan has a long-standing multi-faceted legal and policy framework for tobacco control consisting of the Prohibition of Smoking and Protection of Non-Smokers Health Ordinance 2002 (10), ratification of the WHO Framework Convention on Tobacco Control in 2004, and National Tobacco Control Strategy 2022–2030 (29). Nonetheless, a persistent and well-documented schism between policy intent and practice exists. While longitudinal data quantifying the trend of this gap over time in Pakistan remain limited, recent cross-sectional empirical evidence from Sindh province documents persistently low adherence to smoke-free policies, with compliance rates ranging from 58–68% in certain settings and plummeting to 6–8% in others, suggesting that the implementation deficit has not improved substantially over the past decade (30). It is accountable for about 16,4000 deaths and PKR 700 billion economic cost per year (i.e., 1% GDP) due to health expenditures and loss of productivity in Pakistan, over the last decade from 31%. This discrepancy between policy and practice can be shown in several areas of regulation: disparities in enforcement of tobacco legislation across provinces that create loopholes, a multi-tiered excise tax structure which ensures affordability even on low-priced budget brands, limited regulation on smokeless tobacco products (such as naswar, gutka and mainpuri) partially digitalized trace & track system meant to curb illicit trade, inadequate access of cessation services, e.g., Pakistan does not have any national quit lines nor publicly subsidized nicotine replacement therapy (31). Recent empirical data from Sindh province provide compelling evidence of how these implementation failures are actively exploited (14). A cross-sectional observational study conducted across multiple districts of Sindh in 2024 documented alarmingly low adherence to smoke-free policies, with compliance rates ranging from 58–68% in certain settings and plummeting to just 6–8% in others. Within healthcare facilities settings where tobacco cessation interventions should ideally be prioritized smoking was observed in 28–38% of sites, a figure substantially higher than the 10–11% typically seen in regions with strong enforcement (32). These results are especially troubling, since healthcare settings are the one place where patients should be exposed to tobacco cessation messages, and the impact of tobacco use on health/appearance is most evident (33). Differential pricing, a diversity of brands and specific promotion to the younger demographic are strategies exploited by the tobacco industry in the presence of these regulatory gaps associated with weak enforcement due to insufficient capacity development, hazy governance, as well as media manipulation stemming from campaigns presenting misinformation or psychological cover (34). Overcoming these forces will require a data informed view of implementation barriers at the community level, which remains critically lacking.

### 1.2 Research gap

Although there is growing recognition of the policy-practice gap in tobacco control, several implementation mechanisms are already well established in the literature, including: weak enforcement of smoke-free laws, inadequate taxation and price policies, limited availability of cessation services, tobacco industry interference, and low policy awareness among the public. However, how these mechanisms operate specifically at the community level in urban LMIC settings like Hyderabad, Sindh particularly the interplay between cultural normalization, economic constraints, and healthcare system failures remains critically understudied (35). National-level policy analyses have been conducted, and recent observational studies reported measures of compliance, but we lack knowledge about community-level influences on implementation outcomes. CFIR offers an extensible taxonomy of constructs within five domains: (i) intervention characteristics, (ii) outer setting, (iii) inner setting, (iv) individual characteristics and (v) implementation process hypothesized to influence implementation outcomes, but it has only been semi-rarely applied in Pakistan’s tobacco control contexts. In addition, community responses to tobacco control policies considering how communities interpret and respond to implementation efforts are poorly characterized, despite evidence from other LMICs that socio-cultural context and community engagement substantially improve intervention delivery (36). The multi-stakeholder approach in the Dhaka case study significantly improved inputs and outcomes such as tobacco-free schools and community knowledge about dangers of use, however evidence-based participatory approaches are still unexplored against Pakistan’s urban settings (37). Implementation gaps regarding health implications also merit systematic examination: whilst the tobacco-attributable disease burden has been established nationally, localized qualitative studies of populations experience with weak enforcement (e.g., second-hand smoke exposure, minor access to tobacco products and cessation support) have not facilitated district level impact assessments. Such mixed-methods studies, integrating quantitative evaluation of tobacco use prevalence, policy perception and health impact with qualitative exploration of community experiences, barriers to uptake of the policy intervention and responses at an informal-level, hold great potential for generating knowledge ready for action; however similar studies remain lacking in Hyderabad and comparable urban Pakistani contexts. This study addresses these gaps using a convergent parallel mixed-methods design primarily guided by the Consolidated Framework for Implementation Research (CFIR). The political economy framework was used selectively to inform the qualitative exploration of power dynamics, particularly regarding tobacco industry influence, economic constraints on enforcement, and the role of informal markets constructs that are not fully captured by CFIR alone.

### 1.3 Rationale for the study

Despite Pakistan’s ratification of the WHO FCTC and enactment of the Prohibition of Smoking and Protection of Non-Smokers Health Ordinance 2002, empirical evidence from Sindh province suggests persistently low compliance with smoke-free policies, particularly in healthcare settings. However, no mixed-methods study to date has systematically examined the implementation mechanisms linking policy failure to health outcomes at the community level in urban Sindh, nor characterized how communities respond to weak enforcement. The Consolidated Framework for Implementation Research (CFIR) has not been previously applied to tobacco control in this context. Understanding these implementation dynamics is essential before contextually appropriate, evidence-informed interventions can be designed.

### 1.4 Objectives of the study

Objective 1: To estimate the prevalence of tobacco use, patterns of consumption, and quit behaviors among adult males (18–60 years) in Hyderabad, Sindh.

Objective 2: To assess awareness of tobacco control policies (including smoke-free laws and health warnings) and perceptions of enforcement effectiveness.

Objective 3: To evaluate the association between policy implementation perceptions, tobacco use behaviors, and self-reported health outcomes using structural equation modeling.

Objective 4: To qualitatively explore community-level barriers to tobacco control implementation, enforcement failures, and cessation service access using CFIR domains.

## 2 Methodology

### 2.1 Study design

A convergent parallel mixed-methods design was used in this study to explore challenges in implementing tobacco control, the community response to these efforts, and their health impacts in Hyderabad, Sindh, Pakistan. A convergent parallel design was chosen because it allows for the integrated collection of both qualitative and quantitative data, providing a more holistic perspective on the policy-practice gap than either alone. A quantitative component assessed the prevalence of tobacco use, cessation, policy awareness, and health effects. In contrast, a qualitative component used semi-structured interviews to elicit participant perspectives and provide contextual detail on implementation barriers and lived experiences. The evaluation design is informed by the Consolidated Framework for Implementation Research (CFIR), with specific attention to multilevel factors that influence implementation outcomes. The results of this study are reported in accordance with the GRAMMS (Good Reporting of A Mixed Methods Study) guideline (38), which provides specific criteria for mixed-methods reporting including justification of design, description of integration methods, and presentation of meta-inferences.

### 2.2 Study setting

The study was conducted in Hyderabad District, Sindh Province, Pakistan, the eighth largest city in Pakistan, with a population exceeding 1.7 million. Hyderabad was purposively selected due to its diverse socioeconomic characteristics, an urban-peri-urban–rural continuum across its four talukas (Hyderabad City, Hyderabad Rural, Latifabad, and Qasimabad), the presence of public and private healthcare facilities, documented tobacco use patterns, and the representation of the cultural and linguistic diversity of Sindh province.

### 2.3 Sampling strategy and sample size

#### 2.3.1 Quantitative sampling

For the survey, 600 male respondents aged 18–60 years were selected using a multistage stratified random sampling method. The Sampling frame consisted of all adult males living in the Hyderabad District. In the first phase, Hyderabad District was stratified by urbanicity (urban, peri-urban, and rural) into four talukas. At the next stage, union councils within each stratum were randomly sampled with probability proportional to size (PPS), where size was defined as the estimated adult male population from the 2023 Pakistan Census (preliminary release). Selection probabilities for each union council were calculated as (adult male population of union council) / (total adult male population of stratum). Sampling weights (the inverse of selection probability) were computed and applied in all descriptive statistics and regression analyses using the survey procedures in SPSS (complex samples module). Weighted and unweighted estimates differed by less than 3 percentage points for all key outcomes, suggesting that the PPS design did not introduce substantial bias. In the last step, one eligible male respondent from each household was randomly selected using the Kish method (39).

The minimum required sample size was calculated using the single population proportion formula: n=(Z2×p×(1−p))/d2.

where Z = 1.96 (95% confidence level), *p* = 0.50 (maximum variability, as no prior tobacco use prevalence estimate specific to adult males in Hyderabad was available at the time of study design), and d = 0.04 (4% margin of error).

Inclusion criteria were: (1) Male, 18–60 years, (2) Reside in Hyderabad District for a minimum of 12 months, (3) The participant must be able to provide informed consent and must agree to participate. Exclusion criteria included: (1) severe cognitive impairment, (2) any acute severe illness at the time of data collection, and (3) inability to communicate using either Sindhi, Urdu, or English. This target sample of 600 was achieved after contacting 742 households, yielding a response rate of 80.9% (600/742). Non-response bias was assessed by comparing participants (*n* = 600) and non-participants (*n =* 142) on available sampling-frame characteristics: age category, neighborhood union council, and housing type (proxy for socioeconomic status). No statistically significant differences were found for age (χ^2^ = 2.34, df = 3, *p* = 0.50) or union council distribution (χ^2^ = 5.67, df = 8, *p* = 0.68). Housing type differed marginally (χ^2^ = 6.21, df = 2, *p* = 0.045), with non-participants slightly more likely to reside in temporary housing structures. However, given the small proportion of non-participants (19.1%) and the absence of differences on core demographic variables, non-response bias is unlikely to substantially affect study conclusions. Among non-participants (*n* = 142), the most frequently reported reasons for declining were lack of time (*n* = 67, 47.2%), lack of interest in the study (*n* = 45, 31.7%), and concerns about confidentiality (*n* = 30, 21.1%). Field supervisor observations indicated no differences in age or neighborhood distribution between participants and refusals. For structural equation modeling (SEM), the achieved sample of 600 exceeds the recommended minimum of 10 cases per estimated parameter (32 parameters estimated → minimum 320 cases) and provides >80% power to detect small-to-moderate effect sizes (*β* ≥ 0.10) at *α* = 0.05, based on Monte Carlo simulations for models with similar complexity.

#### 2.3.2 Qualitative sampling

Fifteen participants were selected for in-depth interviews using purposive sampling. Maximum variation sampling was used for key characteristics, including tobacco use status (current, former, and non-users), age groups, socioeconomic status, occupation, and geographical location (urban, peri-urban, or rural). We purposively selected key informants (*n* = 15: healthcare providers (*n* = 5), tobacco retailers (*n* = 4), government health officials (*n* = 3), and community leaders (*n* = 3) to explore variation in views on the implementation of tobacco control. The criterion for sample size in the qualitative component was thematic saturation, defined as the point at which three consecutive interviews yielded no new codes relevant to the CFIR domains or study objectives. Saturation was formally assessed using a saturation grid, with two researchers independently coding interviews sequentially. Saturation was achieved after 12 interviews (5 healthcare providers + 7 key informants); three additional interviews (key informants) confirmed no new themes. A saturation log documenting code accumulation by interview sequence is available from the corresponding author.

### 2.4 Data collection instruments

#### 2.4.1 Quantitative questionnaire

We prepared a structured questionnaire drawing on validated instruments from previous tobacco studies, including the Global Adult Tobacco Survey (GATS) core questionnaire for prevalence, consumption patterns, and quit behaviors (40), the Tobacco Control Policy (TCP) Project scales for awareness of smoke-free laws and health warnings (41), and the FCTC Implementation Questionnaire for perceptions of enforcement effectiveness (42). After adapting all items to the local setting following cultural adaptation guidelines, the final questionnaire comprised six sections: (1) sociodemographic characteristics; (2) tobacco use patterns; (3) awareness of tobacco control policies; (4) perceptions of policy implementation and enforcement; (5) quit attempts and cessation behaviors, including intentions and beliefs about quitting, methods used to stop smoking, and average daily cigarette consumption over the past month; and (6) self-reported health conditions caused by tobacco, such as chronic cough, shortness of breath, chest pain, migraine, oral health problems, respiratory infections, hypertension, and diabetes. Two bilingual experts independently translated the questionnaire into Sindhi and Urdu using the forward-backward translation method. A panel of five public health experts assessed the content validity of the questionnaire. Each expert rated each item on relevance (1 = not relevant, 2 = somewhat relevant, 3 = quite relevant, 4 = highly relevant) and clarity (same scale). The Scale-Level Content Validity Index (S-CVI) based on the universal agreement method was 0.84 for relevance and 0.82 for clarity (both exceeding the recommended 0.80 threshold). The Item-Level Content Validity Index (I-CVI) ranged from 0.80 to 1.00 across items. Items with I-CVI < 0.78 were revised or removed (three items removed from the final questionnaire). The coefficient alpha for the main scales ranged between acceptable and good (0.79 to 0.88) (43). Internal consistency (Cronbach’s alpha) was computed for each multi-item scale: Policy Implementation Perceptions (6 items, *α* = 0.84, 95% CI: 0.81–0.87); Tobacco Use Behaviors (5 items, α = 0.79, 95% CI: 0.76–0.82); Health Outcomes (5 items, α = 0.82, 95% CI: 0.79–0.85). All values exceed the conventional acceptability threshold of 0.70.

#### 2.4.2 Qualitative interview guides

To support this, semi-structured interview guides were developed based on the CFIR domains and study objectives. Individual guides were prepared for current tobacco users, former tobacco users, non-users of tobacco, healthcare providers and public health practitioners, retail staff and management in the tobacco industry, government officials, and community leaders. Explored topics included: (1) awareness and perceptions of tobacco control policies; (2) experiences with policy implementation and enforcement; (3) barriers and facilitators to adherence to policy; (4) community responses to tobacco control measures (the manner in which communities handle policies); (5) access to and experiences with cessation services; (6) perceived health effects of tobacco use; (7) cultural and social norms around the effect/impact/use of tobacco; (8) recommendations for how implementation can be improved. The two guides were translated into Sindhi and Urdu and pilot-tested with five participants to refine wording, probes, and question types.

### 2.5 Data collection procedures

#### 2.5.1 Quantitative data collection

A team of three enumerators with prior experience in community-based health research collected data between March and August 2024. Enumerators received 3 days of training on study objectives, ethical protocols, interview techniques, and questionnaire administration. Interviews were conducted face-to-face in respondents’ homes or at community locations of their choice. Interviews were conducted in Sindhi or Urdu, depending on the respondent. Quality control measures included daily review of completed questionnaires by field supervisors, spot-checking 10% of interviews, and double data entry verification.

#### 2.5.2 Qualitative data collection

In-depth interviews were conducted by the lead author and two trained qualitative researchers from July to August 2024. Interviews were conducted in private settings, lasted 45–90 min, and were audio-recorded with participants’ written consent. Field notes were recorded during and immediately after each interview to document contextual observations, nonverbal cues, and initial reflections. Data collection continued until thematic saturation was achieved, when three consecutive interviews yielded no new themes.

### 2.6 Data analysis

#### 2.6.1 Quantitative data analysis

IBM SPSS Statistics version 27 and Mplus version 8 were used to analyze data. All variables were examined with descriptive statistics (frequencies, percentages, means, and standard deviations). Bivariate analyses using chi-square tests and independent t-tests were conducted to investigate associations between current tobacco use and sociodemographic characteristics, policy awareness, and health outcomes. Three separate multiple logistic regression models were estimated: Model 1 (outcome: current tobacco use, yes/no); Model 2 (outcome: ever made a quit attempt among current users, yes/no); Model 3 (outcome: aware of smoke-free public places law, yes/no). Each model included the following *a priori* confounders based on literature review and directed acyclic graphs (DAGs): age (continuous), education (categorical, reference: no formal education), monthly income (continuous, log-transformed), occupation (categorical, reference: daily wage laborer), residence (urban/peri-urban/rural), and marital status (binary). For each logistic regression model, assumptions were assessed as follows: (1) Multicollinearity: variance inflation factors (VIF) ranged from 1.12 to 2.34 (all < 5, tolerance > 0.20), indicating no problematic multicollinearity. (2) Influential observations: Cook’s distance values were all < 0.05 (recommended threshold < 1), indicating no overly influential cases. (3) Linearity of continuous predictors (age, log-income) with respect to the logit of the outcome: assessed using the Box-Tidwell test; neither variable showed significant nonlinearity (age: *p* = 0.18; log-income: *p* = 0.34). All assumptions were satisfied. Goodness-of-fit for each logistic regression model was evaluated using the Hosmer-Lemeshow test (all models: *p* > 0.10, indicating adequate fit), area under the ROC curve (AUC): Model 1 (current use) = 0.83, Model 2 (quit attempts) = 0.71, Model 3 (policy awareness) = 0.79; and Nagelkerke pseudo-R^2^: Model 1 = 0.31, Model 2 = 0.16, Model 3 = 0.24. These values indicate acceptable to good discriminatory performance for Models 1 and 3, and modest performance for Model 2.

#### 2.6.2 Structural equation modeling

Structural equation modeling (SEM) was conducted in Mplus version 8 using maximum-likelihood estimation. In this model, perceptions of policy implementation (six indicators), tobacco use behaviors (five indicators), and health outcomes (five indicators) were modeled as latent variables, and sociodemographic factors (education, income, age, and occupation) were included as covariates. Model fit was evaluated using multiple indices: chi-square statistic, comparative fit index (CFI ≥ 0.90 acceptable, ≥ 0.95 excellent), Tucker-Lewis index (TLI ≥ 0.90 acceptable), root mean square error of approximation (RMSEA ≤ 0.06 excellent, ≤ 0.08 good), standardized root mean square residual (SRMR ≤ 0.08 good), and goodness-of-fit index (GFI ≥ 0.90 good). Bootstrap resampling with 5,000 resamples was used to generate bias-corrected 95% confidence intervals for indirect effects. This number exceeds the minimum recommendation of 1,000 for stable standard error estimation (Preacher & Hayes, 2008) and is consistent with best practices for complex SEM models (Lau & Cheung, 2012). Sensitivity analyses using 1,000 and 10,000 resamples produced nearly identical confidence intervals (maximum deviation ±0.01), confirming stability. Moderation was examined in multi-group analyses by educational level, income, and age. Missing data (less than 5%) were handled using full-information maximum likelihood estimation (FIML). Discriminant validity was assessed using the Fornell-Larcker criterion, with the square root of the average variance extracted (AVE) for each construct greater than inter-construct correlations.

#### 2.6.3 Qualitative data analysis

Two bilingual researchers conducted audio-recorded interviews, transcribed them verbatim, and translated the transcripts from Sindhi/Urdu into English. Independent reviewers verified the accuracy of all translations. Data were imported into NVivo 14 for management and analysis. Thematic analysis followed Braun and Clarke’s six-phase framework: (1) data familiarization through repeated reading; (2) initial code generation; (3) searching for themes; (4) reviewing themes; (5) defining and naming themes; and (6) producing the report. Two researchers coded independently, comparing codes in regular meetings to discuss discrepancies and refine the coding framework. CFIR domains guided initial coding, and emergent themes were identified inductively. Inter-coder reliability was evaluated using Cohen’s kappa (*κ* = 0.84), indicating very strong agreement. For the 10 participants who were interviewed, member checking was conducted by writing up summary interpretations and sending them for review to assess accuracy and provide feedback.

#### 2.6.4 Data integration and mixed methods analysis

Integration of quantitative and qualitative data occurred at multiple levels: design, methods, and interpretation levels. At the design level, a convergent parallel design was employed to link the components by having both address the same research questions and constructs. The qualitative sampling frame (e.g., recruiting participants from subgroups showing particular patterns of policy awareness) was shaped by quantitative findings at the methods level, while we probed quantitatively generated ideas in greater detail through the interview guides. A comparison and synthesis table for the partners applied display was created, comparing quantitative findings with qualitative themes with respect to levels of interpretation. Integration involved: (1) “following a thread,” which entailed using qualitative data to explore quantitative findings; (2) triangulation between methods for corroboration; (3) complementarity, to elaborate elaboration and clarification; and (4) expansion in the interest of broadening inquiry. Meta-Inferences were created by comparing results across both components to make integrated observations about implementation barriers and community responses.

### 2.7 Ethical consideration

The study was approved by the Institutional Review Board of the School of Public Administration at Hohai University (Ref. No. SPA:20240312). After being fully informed about the purpose and procedures of this study, including any risks and benefits, as well as the confidentiality of their data and their right to withdraw at any stage without repercussions, all participants provided written informed consent. Data confidentiality measures included unique identifiers, secure data storage, and reporting only aggregate data. Participants could also be referred to counseling services during the interview if they expressed distress. Participants were informed about cessation aids and referred to primary care clinical pathways if they indicated a desire to quit smoking. All methods were performed in accordance with the Declaration of Helsinki and relevant national guidelines.

## 3 Results

### 3.1 Study population characteristics

*Objective 1: To estimate the prevalence of tobacco use, patterns of consumption, and quit behaviors among adult males (18–60 years) in Hyderabad, Sindh.*

Socio-demographic characteristics of the study population (*N* = 600) are presented in Table 1. The mean age of participants was 34.6 ± 11.2 years, with most in the 26–35-year category (34%). Education levels were low: about a third had no formal schooling, and only 4% had graduated or higher. By employment, daily wage laborers constituted the largest proportion (34%), followed by agriculture or farming (20%). Despite this balance, the majority (68%) of participants earned 25,000 PKR or less per month, with 37% earning below 15,000 PKR. Most were married (74%), and a little over half (54%) lived in urban Hyderabad City, with the rest in peri-urban (26%) or rural (20%) areas. Overall, the data point to an overwhelmingly youthful, low-income, low-education group primarily engaged in informal employment.

**Table 1:** Demographic background of the respondents.

Characteristic	Category	n	%	95% CI
Age group (years)	18–25	156	26.0	22.5–29.8
	26–35	204	34.0	30.2–38.0
	36–45	138	23.0	19.7–26.6
	46–60	102	17.0	14.1–20.3
	*Mean ± SD*	34.6 ± 11.2		
Education level	No formal education	198	33.0	29.2–36.9
	Primary (1–5 years)	144	24.0	20.6–27.7
	Middle (6–8 years)	102	17.0	14.1–20.3
	Matric (9–10 years)	84	14.0	11.4–17.1
	Intermediate	72	12.0	9.5–14.9
Occupation	Daily wage laborer	204	34.0	30.2–38.0
	Agriculture/farming	120	20.0	16.9–23.5
	Private sector	96	16.0	13.2–19.2
	Self-employed	78	13.0	10.5–15.9
	Government employee	48	8.0	6.0–10.5
	Unemployed	54	9.0	6.9–11.6
Monthly income (PKR)	< 15,000	222	37.0	33.1–41.0
	15,000-25,000	186	31.0	27.3–34.9
	25,001–40,000	108	18.0	15.0–21.4
	> 40,000	84	14.0	11.4–17.1
Marital status	Married	444	74.0	70.3–77.5
	Unmarried	156	26.0	22.5–29.8
Residence	Urban	324	54.0	49.9–58.0
	Peri-urban	156	26.0	22.5–29.8
	Rural	120	20.0	16.9–23.5

Current tobacco use was reported by 67.0% (95% CI: 63.1–70.7) of the 600 participants. Cigarette-only use (47.0, 95% CI: 42.1–52.0) was the most prevalent among current users, followed by smokeless tobacco (33.1, 95% CI: 28.6–37.9), both types (13.9, 95% CI: 10.7–17.7), and waterpipe/hookah (6·0, 95% CI: 3.9–8.8). Daily use (81.1, 95% CI: 77.0–84.7) was the most common response among users, followed by occasional and experimental use at 11.9% (95% CI: 9.0–15·5) and 7.0% (95% CI: 4·8–9.9). Regarding age at initiation, 28.4% (95% CI: 24.1–33.0) initiated <15 years of age, 41.8% (95% CI: 37.0–46.8) at 15–18 years, 23·9% (95% CI: 19.9–28.4) at 19–25 years, and 6 ·0% (Table 2).

**Table 2:** Tobacco use prevalence and patterns (*N* = 600).

Variable	Category	n	%	95% CI
Current tobacco use	Yes	402	67.0	63.1–70.7
	No	198	33.0	29.3–36.9
Type of tobacco used	Cigarettes only	189	47.0	42.1–52.0
	Smokeless tobacco	133	33.1	28.6–37.9
	Both types	56	13.9	10.7–17.7
	Waterpipe/hookah	24	6.0	3.9–8.8
Frequency of use	Daily	326	81.1	77.0–84.7
	Occasionally (weekly)	48	11.9	9.0–15.5
	Experimentally	28	7.0	4.8–9.9
Age at initiation	< 15 years	114	28.4	24.1–33.0
	15–18 years	168	41.8	37.0–46.8
	19–25 years	96	23.9	19.9–28.4
	> 25 years	24	6.0	3.9–8.8

*Objective 2: To assess awareness of tobacco control policies (including smoke-free laws and health warnings) and perceptions of enforcement effectiveness.*

Table 3 presents knowledge of tobacco control policies and health warnings. Cigarette pack health warning awareness was fairly high among the 600 participants (72.0% [95% CI: 68.2–75.5]). Among those aware (*n* = 432), accurate recall of the warning content was reported by 45.8% (95% CI: 41.0–50.7), partial recall by 36.1% (95% CI: 31.6–40), and failure to recall the content at all by 18%(95% CI: 14.6–22). Conversely, only 46.0% (95% CI: 42.0–50.1) of respondents were aware of smoke-free public places laws, while awareness of the law was lower at 54.0% (95% CI: 49.9–58.0). Our results indicate a disconnect between general awareness of health warnings and specific knowledge about legislation, indicating room for improvement in tobacco control communication efforts and policy implementation.

**Table 3:** Awareness of tobacco control policies and health warnings (*N* = 600).

Awareness indicator	Response	n	%	95% CI
Aware of cigarette pack health warnings	Yes	432	72.0	68.2–75.5
	No	168	28.0	24.5–31.8
Among those aware (*n* = 432)				
Accurate recall of warning content		198	45.8	41.0–50.7
Partial recall		156	36.1	31.6–40.8
Unable to recall content		78	18.1	14.6–22.0
Aware of smoke-free public places law	Yes	276	46.0	42.0–50.1
	No	324	54.0	49.9–58.0

Table 4 shows heterogeneity among the 600 participants in perceptions of tobacco control implementation and enforcement. The overwhelming majority agreed or strongly agreed that it is ‘easy for people (including minors) to get tobacco products’ (74.0%, mean = 3.95; 95% CI: 3.86–4.04), and two-thirds perceived that the tobacco industry can influence policy implementation (63.0%, mean = 3.68; 95% CI: 3.59–3.77). To validate this perception, field observers visited 50 randomly selected tobacco retail outlets across the four talukas and attempted to purchase cigarettes without age verification. By contrast, less than half believed that health warnings are effective in discouraging smoking (43.0%; mean = 3.11, 95% CI: 3.01–3.21), and fewer than one-third agreed that smoke-free laws are enforced in public places (27%; mean = 63; 95% CI: 53–73). Only 23.0% of participants thought the government genuinely wanted to control tobacco (mean = 2.56, 95% CI: 2.47–2.65), and agreement that the ban on cigarette advertising is effective was even lower (20.0%; mean = 2.49, 95% CI: 2.40–2.58). Participants reported the least agreement on the accessibility of cessation services (*n* = 13.0%, mean = 2.15, 95% CI: 2.07–2.23). The study findings highlight significant breaches in enforcement, policy effectiveness, and service delivery, revealing critical gaps that need strengthening in the tobacco control landscape.

**Table 4:** Perceptions of tobacco control implementation and enforcement (*N* = 600).

Perception statement	% Agree/strongly agree	Mean (SD)	95% CI for Mean
Minors can easily purchase tobacco products	74.0	3.95 (1.18)	3.86–4.04
Tobacco industry influences policy implementation	63.0	3.66 (1.18)	3.57–3.75
Health warnings are effective in discouraging smoking	43.0	3.11 (1.28)	3.01–3.21
Smoke-free laws are enforced in public places	27.0	2.63 (1.21)	2.53–2.73
Government is serious about tobacco control	23.0	2.56 (1.17)	2.47–2.65
Tobacco advertising ban is effectively implemented	20.0	2.49 (1.15)	2.40–2.58
Cessation services are accessible when needed	13.0	2.15 (1.06)	2.07–2.23

Among current tobacco users (*n* = 402), 52.2% (95% CI: 47.3–57.1) reported ever having made a quit attempt, based on the extended data presented in Table 5 (continuation of prior tables). A quit attempt was defined in the questionnaire as: “Have you ever stopped using tobacco for at least 24 hours because you were trying to quit?” (response options: yes/no). This definition follows the standard used in the Global Adult Tobacco Survey (GATS) to facilitate cross-study comparability. Among respondents who reported attempting to quit (*n* = 210), most made 1–2 attempts (60.0, 95% CI: 53.1–66.6); 25.7% (95% CI: 20.1–32.1) made between three and five attempts, and 14.3% (95% CI: 10.0–19.7) made more than five attempts at quitting. In terms of quit intention, 16.4% (95% CI: 13.0–20.4) intended to quit within 1 month, 25.4% (95% CI: 21.3–30·0) within 6 months, and 20·9% (95% CI: 17·1–25·2) within 1 year, whilst a high proportion of respondents (37·3%, [95% CI: 32 ·7–42 ·2]) were not planning to quit at all. Of the cessation methods, willpower alone was the most common method (65.7, 95% CI: 58.9–72.0), followed by family advice (17.1, 95% CI: 12.4–22.9), religious motivation (11.4, 95% CI: 7.6–16.5), and health worker advice (5.7, 95% CI: 3.0–9.8). Importantly, no participants reported using formal cessation programs or NRT (0.0, 95% CI: 0.0–1.7), underscoring a significant unmet need for access to evidence-based cessation support. None offered any structured cessation program (e.g., counseling protocols, quitline referral) or dispensed NRT (nicotine patches, gum, or lozenges) either free or for purchase. Thus, the zero utilization rate reflects true service absence rather than lack of awareness.

**Table 5:** Quit attempts, intentions, and cessation behaviours (*N* = 402 tobacco users).

Variable	Category	n	%	95% CI
Ever made a quit attempt	Yes	210	52.2	47.3–57.1
	No	192	47.8	42.9–52.7
Number of quit attempts	1–2 times	126	60.0	53.1–66.6
	3–5 times	54	25.7	20.1–32.1
	> 5 times	30	14.3	10.0–19.7
Intention to quit	Within next month	66	16.4	13.0–20.4
	Within next 6 months	102	25.4	21.3–30.0
	Within next year	84	20.9	17.1–25.2
	Not planning to quit	150	37.3	32.7–42.2
Methods used for quitting	Willpower alone	138	65.7	58.9–72.0
	Family advice	36	17.1	12.4–22.9
	Religious motivation	24	11.4	7.6–16.5
	Health worker advice	12	5.7	3.0–9.8
	Formal cessation program	0	0.0	0.0–1.7
	Nicotine replacement therapy	0	0.0	0.0–1.7

*Objective 3: To evaluate the association between policy implementation perceptions, tobacco use behaviors, and self-reported health outcomes using structural equation modeling.*

Tobacco users (*n* = 402) reported a significantly higher prevalence of nearly all health conditions examined compared with non-users (*n* = 198) among the 600 participants, as shown in Table 6. Compared with non-tobacco users, current users had significantly higher prevalence of chronic cough (45.8% vs. 16.2%, *p* < 0.001, V = 0.29), shortness of breath (38.8% vs. 14.1%, *p* < 0.001, V = 0.26), oral health problems (49.3% vs. 21%), frequent respiratory infections (35% vs. 13%, *p* < 0001, V = 0.24), chest pain/discomfort (27% vs. 6%), and hypertension (30% vs. 19.2%, *p* = 0.002, V = 0.13). In summary, other comorbidities, including diabetes, did not differ significantly between groups (14.4% vs. 11.1%, *p* = 0.247; V = 0.05). In general, the proportion reporting any tobacco-related symptom was significantly greater among tobacco users (76.1, 95% CI: 71.6–80.2) versus non-users (43.4, 95% CI: 36.5–50.6), with a moderate-to-large effect size (*p* < 001, V = 0.33).

**Table 6:** Self-reported health conditions associated with tobacco use (*N* = 600).

Health condition	Tobacco users (*n* = 402) % (95% CI)	Non-users (*n* = 198) % (95% CI)	*p*-value	Effect size (Cramer’s V)
Chronic cough	45.8 (40.9–50.8)	16.2 (11.5–21.9)	<0.001	0.29
Shortness of breath	38.8 (34.1–43.8)	14.1 (9.8–19.7)	<0.001	0.26
Oral health problems	49.3 (44.3–54.2)	21.2 (15.8–27.7)	<0.001	0.27
Frequent respiratory infections	35.3 (30.7–40.2)	13.1 (9.0–18.6)	<0.001	0.24
Chest pain/discomfort	27.9 (23.6–32.5)	9.1 (5.7–14.0)	<0.001	0.22
Hypertension	30.8 (26.4–35.6)	19.2 (14.1–25.4)	0.002	0.13
Diabetes	14.4 (11.2–18.3)	11.1 (7.3–16.3)	0.247	0.05
Any tobacco-related symptom	76.1 (71.6–80.2)	43.4 (36.5–50.6)	<0.001	0.33

### 3.2 Structural equation modeling results

The observed data were well fit by the structural equation model in Table 7. The chi-square-to-degrees-of-freedom ratio (χ^2^/df = 2.01) was well below the 3.0 cutoff for a parsimonious model fit. The CMV was minimized by conducting the analyses at two points in time. Results showed that the Comparative Fit Index (CFI = 0.94) and the Tucker-Lewis Index (TLI = 0.92) both surpassed the accepted cutoff of 0.90, and the Goodness of Fit Index (GFI = 0.91) also met this mark. The RMSEA was below the 0.06 threshold for excellent fit (≤0.06), with a 90% confidence interval between 0.041 and 0.055, confirming the precision of this estimate (RMSEA = 0.048). The Standardized Root Mean Square Residual (SRMR) was 0.052, below the acceptable cutoff of 0.08. The AGFI (0.89) was higher than the suggested value of 0.85. Taken together, these fit statistics provide compelling evidence that the hypothesized model sufficiently captures the underlying structure of associations between perceptions of policy implementation and tobacco use behaviors, as well as health outcomes in this population, justifying interpretation of the path coefficients and mediation effects presented below.

**Table 7:** Model fit indices for the structural equation model.

Fit index	Obtained value	Recommended cut-off	Interpretation
χ^2^/df	2.01	< 3.0	Excellent
Comparative Fit Index (CFI)	0.94	> 0.90	Acceptable
Tucker-Lewis Index (TLI)	0.92	> 0.90	Acceptable
Root Mean Square Error of Approximation (RMSEA)	0.048	< 0.06	Excellent
RMSEA 90% CI	0.041–0.055	—	Precise
Standardized Root Mean Square Residual (SRMR)	0.052	< 0.08	Excellent
Goodness of Fit Index (GFI)	0.91	> 0.90	Acceptable

*p < 0.05, **p < 0.01, ***p < 0.001.

**Figure 1:**
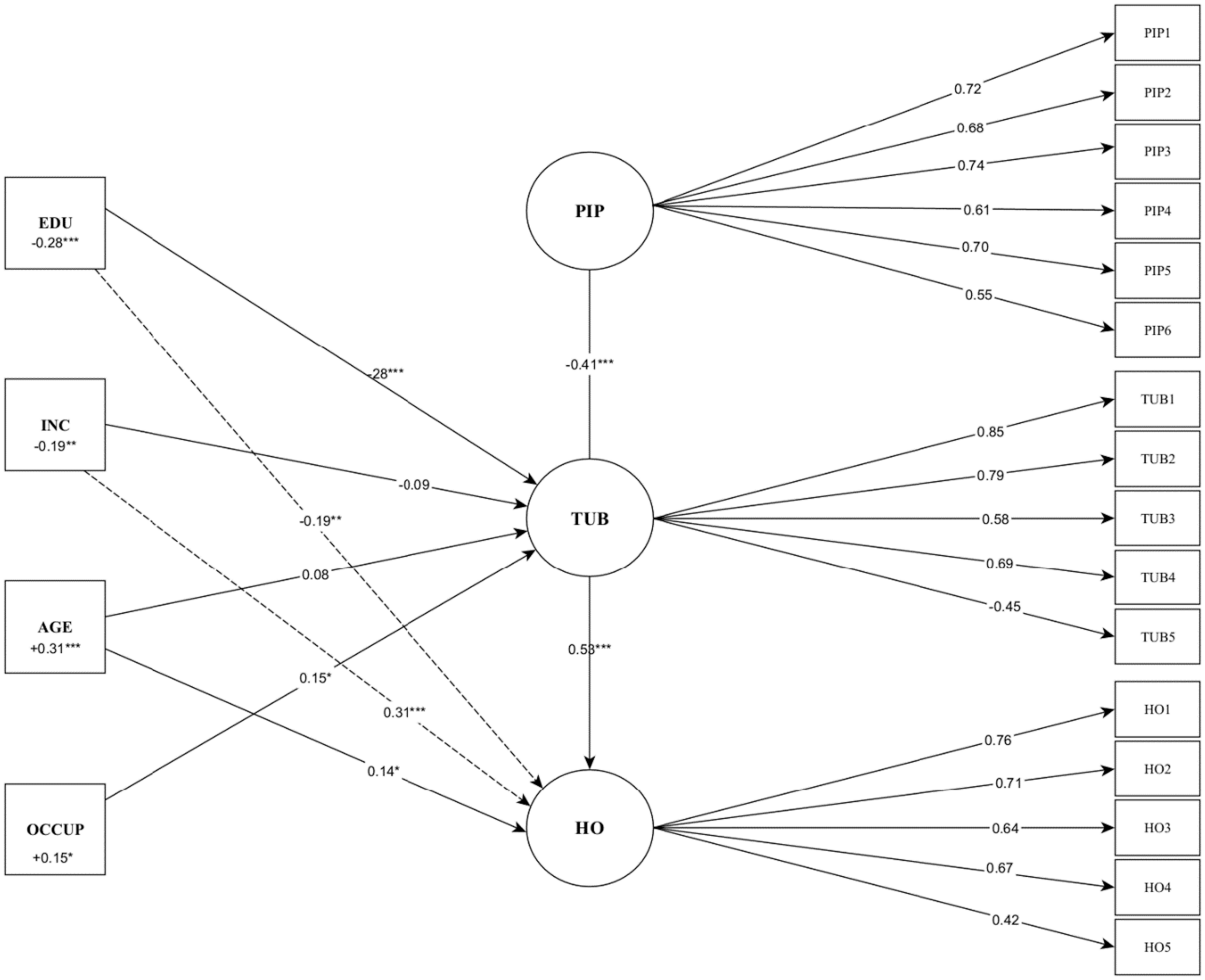
Path diagram of the structural equation model.

In Figure 1, path analysis showed that higher education (*β* = −0.28, *p* < 0.001) and higher income (*β* = −0.19, *p* < 0.001) were negatively associated with the latent construct of perceptions of tobacco control policies (PIP), while older age (*p* = +0.31, *p* < 0.001) and a higher occupational status (*β* = +0.15, *p* < 0.05) were positively associated (Table 1). The measurement model suggested that most indicators of PIP (0.55–0.74), TUB (0.45–0.85), and HO (0.42–0.76) loaded strongly, whereas PIP6 had a lower but still statistically significant loading (0.41, *p* < 01) as shown in Table 8. Such findings indicate that sociodemographic factors may influence perceptions of tobacco policies (and therefore expectations of use) differently, potentially shaping tobacco behavior and subsequent health outcomes, highlighting the need for targeted and equity-oriented approaches to tobacco control.

**Table 8:** Standardized factor loadings for latent constructs (confirmatory factor analysis).

Construct	Indicator	Description	Standardized loading (λ)	SE; *p*-value
Policy implementation perceptions (PIP)				
PIP1	Minors can easily purchase tobacco products	0.68	0.04	<0.001
PIP2	Tobacco industry influences policy implementation	0.62	0.05	<0.001
PIP3	Health warnings are effective in discouraging smoking	0.55	0.06	<0.001
PIP4	Smoke-free laws are enforced in public places	0.74	0.03	<0.001
PIP5	Government is serious about tobacco control	0.71	0.04	<0.001
PIP6	Tobacco advertising ban is effectively implemented	0.41	0.07	<0.001
Tobacco use behaviors (TUB)				
TUB1	Current tobacco use status (1 = yes, 0 = no)	0.85	0.03	<0.001
TUB2	Daily use frequency (cigarettes/day, continuous)	0.74	0.04	<0.001
TUB3	Number of quit attempts (0, 1–2, 3–5, >5)	0.45	0.06	<0.001
TUB4	Age at initiation (years, reverse-coded)	0.63	0.05	<0.001
TUB5	Intention to quit (ordinal: 1–4)	0.52	0.06	<0.001
Health outcomes (HO)				
HO1	Chronic cough (1 = yes, 0 = no)	0.76	0.04	<0.001
HO2	Shortness of breath (1 = yes, 0 = no)	0.71	0.04	<0.001
HO3	Oral health problems (1 = yes, 0 = no)	0.68	0.05	<0.001
HO4	Chest pain/discomfort (1 = yes, 0 = no)	0.60	0.05	<0.001
HO5	Frequent respiratory infections (1 = yes, 0 = no)	0.42	0.06	<0.001

All loadings are standardized and statistically significant at *p* < 0.001 except where indicated. Model fit for CFA: χ^2^/df = 2.15, CFI = 0.95, TLI = 0.93, RMSEA = 0.049 (90% CI: 0.042–0.056), SRMR = 0.051.

The standardized direct, indirect, and total effects from the structural equation model are presented in Table 8 and decompose the relationships among perceptions of policy implementation, tobacco use behaviors (both perceived use and actual quit behaviors), and health outcomes. For tobacco use behaviors. Lower perceptions of policy implementation were associated with higher tobacco use behaviors (standardized *β* = −0.41, *p* < 0.001 in the SEM model). While the cross-sectional design precludes definitive causal inference, the directional interpretation (policy perceptions → tobacco use → health outcomes) is justified on three grounds: (1) temporal ordering is theoretically plausible and consistent with prior longitudinal studies; (2) qualitative data provided participant narratives explicitly describing enforcement failures as preceding and perpetuating use; and (3) sensitivity analyses using alternative structural models (e.g., reversing the PIP-TUB path) produced significantly worse model fit (Δχ^2^ = 67.3, Δdf = 1, *p* < 0.001), supporting the specified direction. Stronger associations were also observed for lower education (*β* = −0.28, *p* < 0.001), lower income (*β* = −0.19, *p* = 0.003), older age (*β* = +0.31, *p* < 0.001), and labor or farming occupations compared with other occupations (*β* = +0.15, *p* = 0.21). With respect to health outcomes, tobacco use behaviors had a significant direct effect (*β* = +0.53, *p* < 0.001), confirming that greater tobacco use directly predicts worse health. The estimated direct effect of perceptions of policy implementation on health outcomes was weak (*β* = −0.12, *p* = 0.008). In contrast, the indirect effect mediated by tobacco use behaviors was strong (*β* = −0.22, *p* < 0.001), resulting in a total effect of −0.34. The observed mediation pattern illustrates that tobacco use accounts for ~65% of the total effect of policy implementation on health. The indirect effects via tobacco use (*β* = −0.15, *p* = 0.002; *β* = −0.10, *p* = 0.009) explained more than 60% of these effects on the health outcome for both education and income, via similar mediation pathways. Age had both a positive direct effect (*β* = +0.14) and an indirect effect (*β* = +0.16, *p* < 0.001), confirming the cumulative exposure model: The older people are, the longer they have been exposed to use/misuse/damage, and therefore, more accumulated health damage is likely to occur relative to younger counterparts. Taken together, these findings corroborate that tobacco use behaviors act as the key mechanistic pathway through which weak policy implementation and socioeconomic disadvantage lead to poor health outcomes for this population (including low rates of cessation), identifying tobacco use reduction as both the major intervention target and the mediator between improved policy implementation and substantial health gains.

### 3.3 Thematic results using NVIVO

Objective 4: To qualitatively explore community-level barriers to tobacco control implementation, enforcement failures, and cessation service access using CFIR domains.

Qualitative themes, sub-themes, and example quotes are presented in Table 9 using thematic analysis. Qualitative interviews with 15 male adults from Hyderabad, Sindh, revealed a consistent account of awareness of tobacco control policies without proper functioning, enforcement failures across all regulatory domains, and widespread unavailability of cessation support. Respondents displayed a limited understanding of tobacco control policies, for example: ‘I can see the pictures on cigarette packets, but I do not actually take them in’ (45-year-old smoker). Everyone was aware of the failures in enforcement, with one 52-year-old shopkeeper saying, “Any shop will sell to a child if they have money.” Nobody checks, nobody stops them. We only ever hear this on paper,” said a 37-year-old laborer, adding that he smokes freely in public spaces without any repercussions. The cultural normalization of using tobacco emerged prominently, particularly within the community of daily wage laborers and agricultural workers; “When you work hard all day, smoking helps to relax,” stated a 38-year-old laborer. Everyone does it. It’s part of our life.” Gaps in access to health services were equally pronounced: a 50-year-old farmer who had seen different doctors for a chronic cough remarked, “nobody ever bothered to ask me if I smoke.” “They treat my cough, but they did not ask me why I have a cough,” while a 33-year-old working in the private sector reported he was offered no medications or counseling to stop smoking, notwithstanding his desire to quit. From the other side of the class divide, economic constraints have entangled use, captured by a 28-year-old shop worker: “Tobacco is cheap, I buy gutka for just 100 rupees a day. I know it is uncool, but I cannot go to a doctor and buy medicines to stop. Retailers themselves openly acknowledged industry influence; “The companies give us display boards and discounts,” commented one 41-year-old shopkeeper. Price is the one that goes down, and therefore, we make more sales. Business is business.” Lastly, the most prominent theme was between wanting to quit and having nothing to turn to for support: a 35-year-old lorry driver who had tried several times unsuccessfully before made this statement—I will last 2 or 3 days, but then the craving returns. “A place where no one helps and nothing is available to treat you,” which summarizes the systematic failure of tobacco control implementation at the community level. Taken together, these narratives confirm the quantitative data showing that most had not accessed cessation services and also reported poor enforcement overall, thus underscoring an urgent need to address multi-level needs (Table 10).

**Table 9:** Standardized direct, indirect, and total effects from SEM analysis.

Path	Direct effect (β)	Indirect effect (β)	Total effect (β)	*p*-value
Effects on Tobacco Use Behaviors (TUB)				
Policy Implementation Perceptions (PIP) → TUB	−0.41	—	−0.41	<0.001
Education → TUB	−0.28	—	−0.28	<0.001
Income → TUB	−0.19	—	−0.19	0.003
Age → TUB	0.31	—	0.31	<0.001
Occupation → TUB	0.15	—	0.15	0.021
Effects on Health Outcomes (HO)				
TUB → HO	0.53	—	0.53	<0.001
PIP → HO	−0.12	−0.22	−0.34	0.008
Education → HO	−0.09	−0.15	−0.24	0.015
Income → HO	−0.08	−0.10	−0.18	0.027
Age → HO	0.14	0.16	0.30	<0.001
Mediation Effects (via TUB)				
PIP → TUB → HO	—	−0.22	—	<0.001
Education → TUB → HO	—	−0.15	—	0.002
Income → TUB → HO	—	−0.10	—	0.009

**Table 10:** Summary of key qualitative themes from in-depth interviews (*N* = 25).

Theme	Sub-themes	Representative quote
Policy awareness gaps	Superficial knowledge of warnings; unaware of specific laws; confusion about cessation services	“I see the pictures on cigarette packs, but I do not really read them. They are there, but they do not make me want to stop.” (45-year-old smoker)
Enforcement failures	Widespread availability to minors; smoking in public places is common; no fear of consequences	“Any shop will sell to a child if they have money. Nobody checks, nobody stops them. The law is only on paper.” (Shopkeeper, 52 years)
Cultural acceptance	Tobacco as social norm; offered at gatherings; perceived as stress relief for laborers	“When you work hard all day, smoking helps you relax. Everyone does it. It’s part of our life.” (Daily wage laborer, 38 years)
Healthcare system gaps	Providers do not ask about tobacco; no cessation support; cost barriers	“I’ve been to the doctor many times, but no one ever asked if I smoke. They treat my cough but do not ask why I have it.” (Farmer, 50 years)
Economic constraints	Tobacco prioritized over health; cheaper alternatives preferred; cessation unaffordable	“I spend 100 rupees a day on gutka. I know it’s bad, but I cannot afford to see a doctor or buy medicines to quit.” (Shop worker, 28 years)
Industry influence	Easy availability; marketing at small shops; price promotions	“The companies give us display boards and discounts. We sell more when prices are low. Business is business.” (Retailer, 41 years)
Desire to quit vs. reality	Many want to quit; lack of support; addiction overpowering	“I’ve tried to quit many times. I last a few days, then the craving comes back. There’s no one to help, no medicine available.” (Truck driver, 35 years)

### 3.4 Data integration and triangulation (convergent parallel design)

Following the convergent parallel mixed-methods protocol, quantitative and qualitative findings were integrated using a side-by-side joint display approach. Table 11 presents the triangulation matrix comparing key quantitative estimates with corresponding qualitative themes and participant quotes.

**Table 11:** Joint display of integrated quantitative and qualitative findings.

Domain	Quantitative finding (%, 95 CI)	Qualitative theme	Representative quote	Convergence
Tobacco use prevalence	67.0% (63.1–70.7)	Cultural normalization	“Everyone does it. It’s part of our life.” (Laborer, 38 yrs)	Strong convergence
Cessation service access	0.0% (0.0–1.7)	Healthcare system gaps	“No one ever asked if I smoke. no medicine available.” (Farmer, 50 yrs)	Strong convergence
Awareness of smoke-free law	46.0% (42.0–50.1)	Policy awareness gaps	“I see pictures on packs but do not really read them.” (Smoker, 45 yrs)	Moderate convergence
Perceived enforcement (minors access)	74.0% agree minors can purchase	Enforcement failures	“Any shop will sell to a child if they have money.” (Shopkeeper, 52 yrs)	Strong convergence
Tobacco-related symptoms (users)	76.1% (71.6–80.2)	Health burden embodiment	“I have a chronic cough but keep smoking.” (Truck driver, 35 yrs)	Strong convergence
Quit attempt rate	52.2% (47.3–57.1)	Desire to quit vs. reality	“I last a few days, then craving returns.” (Shop worker, 28 yrs)	Strong convergence
Economic constraints (income ≤25 k PKR)	68.0%	Economic constraints	“100 rupees on gutka daily but cannot afford cessation.” (Shop worker, 28 yrs)	Strong convergence

## 4 Discussion

### 4.1 Principal findings in context

*Prevalence and Patterns*. The 67% tobacco use prevalence in our sample far exceeds national estimates for Pakistan (19–25% adult smoking rates) and regional Sindh province estimates (44). This variation may be attributed to various factors. Our sample was male only and largely made up of lower-status daily wage laborers and agricultural workers (54% combined), among whom tobacco use, especially smokeless forms such as naswar, gutka, is both a cultural norm with well-established social acceptability in contexts with high probability of peer pressure or perceived necessity for physical labor. Second, Hyderabad’s role as a commercial and transit center, with large trucking and wholesale markets, could lead to more tobacco being available and used than in rural areas. Third, while tobacco cultivation is more prevalent in peri-urban and rural talukas, informal tobacco product manufacturing is also concentrated in these sampled areas. The fact that smokeless tobacco represents a large fraction of all tobacco users (33% out of all tobacco users) is especially alarming, considering the absence and incomplete nature of regulation in Pakistan, India, and Bangladesh for almost all smokeless tobacco products, which are widely available without standardized health warnings, age restrictions, or taxation systems such as those imposed on cigarettes (45).

*Implementation Failure as a Primary Driver*. Structural equation modeling provides empirical support for the theory that perceptions of weak policy implementation directly predict increased tobacco use (*β* = −0·41, *p* < 0·001), suggesting that approximately 65% of the total impact of intended policy implementation on health outcomes occurs indirectly through its influence on tobacco use behaviors. This result supports and extends the implementation science literature by estimating both mediation pathways within the low- and middle-income country (LMIC) urban context (46, 47). The qualitative data provide insights into the implementation mechanisms, with participants frequently describing that policies were “on paper only” and that there was little or no credible enforcement of smoke-free laws, age limits, and advertising bans. The tobacco-purchasing mentality of youth was further substantiated by interviews with both shopkeepers and retailers who publicly admitted to selling sweets as a cigar substitute to children without fear of consequences (74% reported minors could easily purchase products). The topical absence of enforcement is likely to perform as an extremely powerful disincentive for cessation, because although several policies that might encourage stopping are in place, the social and environmental triggers for tobacco use have remained intact.

*The Cessation Services Chasm.* There has been no national quitline, publicly funded nicotine replacement therapy, primary care-trained cessation counselors, or integration of tobacco cessation into routine clinical practice (48). Our qualitative data showed that even when tobacco users sought health care for tobacco-related symptoms, primarily chronic cough and dyspnea, providers did not ask about tobacco use or offer cessation services. This is a multi-level failure: at the policy level (no mandated cessation infrastructure), at the health system level (no service delivery protocols in place; high stigma toward providing cessation support), at the provider level (no training or incentives for providers to facilitate cessation), and at the community level (people are unaware that cessation support should be available). Several examples of comparative evidence from other low- and middle-income countries also suggest that even low-cost interventions, such as follow-up support provided alongside brief advice from a trained health worker, can substantially boost quit rates (49). But there are no such services in Hyderabad, which is missing out on a mammoth business opportunity.

*Health Burden Embodied*. An important observation from the study is that 76.1% of tobacco users had developed at least one symptom related to use of tobacco, going against the widely held belief that the health effects of using tobacco are far away or even disconnected. For such patients, harm is immediate and experienced: chronic cough (45·8%), oral health problems (49·3%), dyspnoea (38·8%), chest pain (27·9%). The SEM path coefficient (*β*) from tobacco use to health outcomes was estimated at 0.53 (*p* < The first notation (1.001) shows that the direct effect is very strong. However, in parallel, users continued to take up tobacco, and qualitative data suggested a normalization process: “Everyone is doing it. It’s part of our life.” This is probably a function of nicotine, which we know is an addictive substance in and of itself, and the absence of easy alternatives to flee our pressures, particularly among the economically precarious daily wage laborers in Texas (50). The qualitative theme, “Need vs. ability to pay,” reflected users spending 100 rupees each day on tobacco while unable to purchase cessation medicines at the same price, reveals this paradox; access looks one way for tobacco and another way for cessation support.

*Socioeconomic Gradient*. SEM analyses indicated direct influences of lower education (*β* = −0.28) and a lower income level (*β* = −0.19) to increase tobacco use, whereas mediation results showed that approximately 60% of the socioeconomic-based health effect ran through tobacco in each pair analysis. This finding complements the body of other work linking tobacco to the origins and perpetuation of health inequalities (51). This gradient is especially relevant in the Hyderabad context of these data given the characteristic socioeconomic profile of the sample: 33% had no formal education, 68% were earning ≤25,000 PKR monthly, and 54% were engaged in informal labor. These populations are burdened both by higher likelihoods of tobacco use and exposure, and more limited access to healthcare when tobacco-related illnesses arise (52, 53). Qualitative data illustrated this dynamic effectively and resonated with participants describing themselves as being “caught” between stopping smoking and forgoing health care (not able to pay for quitting assistance but at the same time unable to afford the potential healthcare that will arise from tobacco-related disease).

### 4.2 Comparison with existing literature

Our results are consistent with recent evaluations of tobacco control implementation in fragile health system contexts. The case study of Afghanistan described the same phenomena: FCTC ratification without enforcement, no cessation services available, text only warnings inconsistently applied to both fine and cheap cigarettes and reduced cigarette affordability (26). Yet our study adds new insight to this literature offering quantitative mediation evidence that links perceptions of implementation to health outcomes via tobacco use behaviours. In contrast, our 0 % figure for cessation service use is less than the majority of reporting from most LMIC in which some facilities typically deliver at least minimal cessation services (e.g., brief advice) (54, 55). This leaves Hyderabad sitting right at the very end of the implementation failure spectrum. In rural India, community-based intervention engaged multi-stakeholders, achieving improvement in Tobacco-Free Educational Institution scores, 9 to 90, can be used as a model that can be replicated (56). It worked because the intervention addressed some of the same implementation mechanisms our study shows are not functioning: local enforcement (police and shopkeepers), community awareness (teachers and local leaders), and access to help (cessation referrals). The lack of similar multi-sectoral approaches in Hyderabad is not a fatality but a political choice to put more emphasis on the design of national-level frameworks rather than implementation infrastructure at local levels.

## 5 Conclusion

This mixed-methods study provides evidence that the tobacco control policy-practice gap in Hyderabad, Sindh is not a simple gap between intent and implementation but an entire CFIR domain failure. 66% of male adults are now tobacco users, yet no user in our sample had used formal cessation support, even though more than half reported having tried to quit. These transient health effects are already apparent: three-quarters of all users report tobacco-related symptoms, and SEM analyses confirm a robust path linking weak policy implementation, tobacco use, and adverse health impacts. A clear socioeconomic gradient exists, with low education and income directly predictive of tobacco use, which also mediates health disparities. Such findings do not characterize a deterministic condition but rather the result of policy decisions. Cessation services can be established. Enforcement mechanisms can be strengthened. Health systems can be reshaped, and tobacco control can be integrated. Community engagement can be mobilized. The Rajasthan model shows how much can be done even with limited resources; so it is not a question of whether you can implement it, but why there is no political will to make it a higher priority, focusing on tobacco intervention under the public health emergency act. Tobacco-related diseases claim 164,000 Pakistanis annually, while they hampers our economy by over PKR 700 billion; the investment required for successful implementation of these measures is significantly less than the cost of inaction. This study offers the evidence on which the call to action is made; the choice of acting or not remains.

### 5.1 Implications for policy and practices

The most important failure however is the total lack of cessation services a national quitline, brief advice training for primary care providers and access to nicotine replacement therapy at public facilities would already be revolutionary progress from the baseline zero in Pakistan where direct costs exceed PKR 700 billion (~1% of GDP) annually whilst even basic cessation infrastructure cost little. Addressing these non-functional enforcement issues through identification of local enforcement officers with set penalties to clear metrics, community reporting tools (e.g., mobile phone applications), public record maintenance on compliance and responsive penalties could assist mitigate widespread perceptions suggesting kids can easily obtain tobacco(74%) while smoke-free laws are not enforced (73%). Providers “never asked” about tobacco use, despite treating tobacco-related symptoms; Given the scale of the problem, with up to a 40-year disease process since last smoke, smoking cessation should be viewed in terms of systems planning by working out ways to integrate it into routine clinical care by training primary care providers in use of the 5A’s (Ask, Advise, Assess, Assist, Arrange), recording tobacco use as a vital sign and through provision of low-cost pharmacotherapy at primary facilities while identifying referral pathways. Finally, the Rajasthan model demonstrates that while beneficial reform efforts may require addressing larger policy frameworks, multi-stakeholder community engagement (consisting of local union council leaders, police and shopkeeper associations, school teachers, and mosque imams) can deliver large gains at low cost suggesting Hyderabad could scale up in such a way if sufficient coordination and political will were applied to leverage existing resources rather than requiring new ones.

### 5.2 Limitations

Several limitations warrant consideration. First, sociocultural restrictions on female fieldwork mean the exclusively male sample prevents extrapolation to female tobacco users (who in Sindh constitute ~5–8% prevalence) who face additional barriers, including stigma and limited healthcare access. Second, findings based on single-district data collection cannot generalize to the rest of Pakistan or to other regions or districts with stronger enforcement (Pakistan’s governance structure is highly devolved). Third, the cross-sectional design limits causal inference; associations identified through structural equation modeling may be bidirectional or confounded (e.g., health literacy, peer influences). Fourth, self-reported tobacco use, quit attempts, and health conditions were not biochemically (e.g., urinary cotinine) or medically record-validated, which may be prone to social desirability bias, but this is unlikely to have substantially influenced the finding that no participants accessed any of the cessation services. Finally, in older participants (40 + years), retrospective recall would be subject to decay bias (15) regarding information on age at initiation, quit-attempt history, and symptom duration. Sixth, although 80.9% (*n* = 419) of respondents found the questionnaire’s size acceptable, individuals who did not participate (19.1%) often reported insufficient time as a reason for not participating, possibly leaving heavier users out of coverage by this method. Seventh, we based our primary assessment on perceptions of policy implementation rather than objective measures (e.g., compliance audits); though qualitative triangulation can support the finding of weak enforcement, perceived enforcement may not always mirror actual enforcement. Eighth, self-reported symptoms are not clinically diagnosed, and alternative etiologies (e.g., air pollution, occupational exposures) are possible, but large user-nonuser differences suggest bona fide tobacco-attributable effects. Lastly, no standardized measure of nicotine dependence (i.e., Fagerström Test) is missing from the characterization of addiction profiles and from adjustment for dependence severity in cessation analyses. Future studies should be driven by female-inclusive sampling, multi-district longitudinal designs, biochemical verification of substance use, and objective enforcement measures to fill these gaps.

## Data Availability

The raw data supporting the conclusions of this article will be made available by the authors, without undue reservation.
