# Supplementary material for: Bridging the gap between policy and practice: a mixed-methods study of tobacco control implementation challenges and community responses in Hyderabad, Sindh, Pakistan
Source: Front Public Health. 2026 Jun 18;14:1881029. doi: 10.3389/fpubh.2026.1881029 (PMC13323013; doi:10.3389/fpubh.2026.1881029)
Supplement: Supplementary file 1 [file Table_1.DOCX]

**APPENDIX**

**Table-1: GRAMMS Six-Item Checklist**

| # | Criterion | Focus Area | Response (with manuscript references) |
| --- | --- | --- | --- |
| **1** | **Justification for mixed methods** | State the justification for utilising mixed methods in this study | **Response:** A convergent parallel mixed-methods design was justified because tobacco control implementation failures operate at multiple levels (policy, health system, community, individual) that cannot be fully captured by a single method. Quantitative data alone can measure prevalence, awareness, and statistical associations but cannot explain *why* enforcement fails or *how* communities respond to weak policies. Qualitative data alone can provide rich contextual accounts but cannot estimate the magnitude of the implementation gap or quantify the pathway from policy perception to health outcomes. By combining both approaches, this study aimed to: (1) quantify the prevalence of tobacco use, policy awareness, and health effects (breadth); (2) qualitatively explore the mechanisms of implementation failure, cultural normalization, and cessation service gaps (depth); and (3) integrate findings to generate actionable meta-inferences about priority intervention targets. ***(Location: Introduction Section 1.2 Rationale, page 5; Methodology Section 2.1, page 6)*** |
| **2** | **Description of design** | Describe the type, priority and sequence of the different methods of study employed | **Response:** **Type:** Convergent parallel design (also known as concurrent triangulation design). **Priority:** Equal weight given to both strands (QUAN + QUAL), as quantitative and qualitative components were equally essential to address the implementation research questions. **Sequence:** Concurrent collection – quantitative surveys and qualitative in-depth interviews were conducted during the same field period (March to August 2024) with no temporal separation. Within participant subgroups (e.g., current tobacco users), the quantitative survey was administered first, followed by the qualitative interview within 7-14 days, allowing preliminary quantitative analysis to inform probing questions during interviews. **Integration points:** Predetermined at three levels – (1) design (qualitative sampling frame informed by quantitative strata), (2) methods (joint display comparison), and (3) interpretation (meta-inference generation). ***(Location: Methodology Section 2.1, page 6; Section 2.1.1 Protocol Specification, page 7)*** |
| **3** | **Methods detail** | Describe the sampling approach for both quantitative and qualitative data collection and description of data analysis method for both methods | **Response:** **Quantitative sampling:** Multistage stratified random sampling of 600 male adults (18-60 years) from Hyderabad District. First, stratification by urbanicity (urban, peri-urban, rural) across four talukas. Second, random sampling of union councils with probability proportional to size. Third, random household selection using random number generation. Fourth, Kish method for selecting one eligible male per household. Sample size calculated using single proportion formula: n = (Z² × p × (1-p))/d² = (1.96² × 0.50 × 0.50)/0.04² = 600; plus 15% non-response adjustment. **Qualitative sampling:** Purposive maximum variation sampling of 25 participants, including current tobacco users (n=10), former users (n=5), non-users (n=5), healthcare providers (n=5), tobacco retailers (n=4), government officials (n=3), and community leaders (n=3). Sample size determined by thematic saturation (three consecutive interviews with no new themes). **Quantitative analysis:** Descriptive statistics (frequencies, means, 95% CIs), bivariate analyses (χ² tests, t-tests), multiple logistic regression, and structural equation modeling (SEM) in Mplus v8 with maximum likelihood estimation. Model fit evaluated using CFI, TLI, RMSEA, SRMR, GFI. **Qualitative analysis:** Thematic analysis following Braun & Clarke's six-phase framework in NVivo 14. Initial coding guided by CFIR domains, with inductive identification of emergent themes. Independent double coding with Cohen's κ=0.84, member checking (n=10), and reflexivity practices. ***(Location: Methodology Section 2.3, pages 7-8; Section 2.6, pages 10-11)*** |
| **4** | **Integration** | State how the quantitative and qualitative data was integrated | **Response:** Integration occurred at four levels using multiple techniques: **(1) Design-level integration:** The qualitative sampling frame was shaped by quantitative findings (e.g., recruiting participants from subgroups showing particular patterns of policy awareness). Interview guides were developed to probe quantitatively identified patterns (e.g., the 0% cessation service access, 74% perception of weak enforcement). **(2) Methods-level integration (following a thread):** Quantitative variables (policy awareness scores, tobacco use frequency) were transformed into ordinal categories (low/medium/high) to facilitate side-by-side comparison with qualitative themes using a joint display table (Table 10 in Results Section 3.5). **(3) Triangulation:** Quantitative findings (e.g., 67% prevalence, 0% cessation access, 76% with tobacco-related symptoms) were directly compared with qualitative themes (cultural normalization, healthcare system gaps, health burden embodiment) to assess convergence, complementarity, or divergence. **(4) Interpretation-level integration (meta-inferences):** Integrated conclusions were generated that go beyond separate findings – three overarching meta-inferences about implementation failure, enforcement weakness, and the socioeconomic gradient. ***(Location: Methodology Section 2.6.4, page 11; Results Section 3.5 with Table 10, pages 27-28)*** |
| **5** | **Method limitations** | Identify the limitations associated with combining the two methods used for the study (mixed methods) | **Response:** Specific limitations of the mixed-methods approach include: **(1) Integration challenges:** Although no divergent findings were identified, integrating different data types required data transformation (quantitizing qualitative themes for joint display), which may oversimplify rich qualitative narratives. **(2) Sampling asymmetry:** The quantitative sample (n=600) was much larger than the qualitative sample (n=25), creating potential imbalance in the weight given to each strand despite equal priority designation. **(3) Temporal separation:** While data collection was concurrent, the 7-14 day gap between survey and interview for the same participants may have allowed intervening experiences to influence qualitative responses. **(4) Generalization limitations of integrated findings:** Meta-inferences derived from a single district (Hyderabad) with a male-only sample cannot be generalized to women, other districts of Sindh, or other provinces of Pakistan without further validation. **(5) Integration validity threats:** The potential for confirmation bias exists when qualitative findings are used to "explain" quantitative results – the research team mitigated this through independent double coding, reflexivity, and member checking. **(6) Resource intensity:** The convergent parallel design required twice the data collection resources and analytical expertise in both quantitative and qualitative methods, which may limit replicability in lower-resource settings. ***(Location: Discussion Section 5.2 Limitations, page 35, with mixed-methods specific limitations added as above – to be inserted as a new paragraph in the limitations section)*** |
| **6** | **Insights from integration** | Provide details of any new knowledge gained through the integration of quantitative and qualitative data | **Response:** Integration of quantitative and qualitative data yielded three novel meta-inferences that could not have been obtained from either method alone: **(1) Absolute cessation service failure:** Quantitative data showed 0.0% (95% CI: 0.0-1.7) of tobacco users accessed formal cessation services despite 52.2% reporting quit attempts. Qualitative data revealed *why* – healthcare providers never asked about tobacco use ("no one ever asked if I smoke" – farmer, 50 years), no referral pathways existed, and participants were unaware that cessation support should exist. Integration demonstrates the failure is not incremental but absolute across all levels of the health system. **(2) Enforcement weakness as behaviorally consequential:** Quantitative data showed 74% believed minors could easily purchase tobacco and 73% believed smoke-free laws were unenforced. Qualitative data revealed these perceptions directly shape behavior – shopkeepers openly admitted selling to children ("any shop will sell to a child if they have money" – shopkeeper, 52 years), and smokers reported no fear of consequences. Integration demonstrates perceived enforcement weakness is not merely a knowledge gap but a behavioral determinant. **(3) Dual affordability pathway of socioeconomic gradient:** Quantitative SEM analysis showed lower income directly predicted tobacco use (β = -0.19, p = 0.003), with 60% of socioeconomic health effects mediated through tobacco use. Qualitative data revealed a paradoxical dual pathway – participants spent PKR 100 daily on tobacco ("I spend 100 rupees a day on gutka" – shop worker, 28 years) while reporting inability to afford cessation medicines ("can't afford to see a doctor or buy medicines to quit"). Integration demonstrates tobacco is perceived as affordable while cessation is not, creating a structural barrier to quitting that is invisible in single-method studies. ***(Location: Results Section 3.5 Meta-inferences, page 28; Discussion Section 4.1, pages 29-31)*** |

**Declaration**: This GRAMMS checklist has been completed to document the reporting quality of the mixed-methods study presented in this manuscript. The study design, conduct, analysis, and reporting followed the principles of good practice for convergent parallel mixed-methods research as articulated by Creswell and Plano Clark (2018) and the GRAMMS guideline (O'Cathain et al., 2008). All items are fully addressed as indicated above.

**Table-2: Summary Table for Quick Reference**

| GRAMMS Criterion | Focus Area | Status | Page Reference |
| --- | --- | --- | --- |
| 1 | Justification for mixed methods | ✓ Completed | Introduction 1.2, Methodology 2.1 |
| 2 | Description of design (type, priority, sequence) | ✓ Completed | Methodology 2.1, 2.1.1 |
| 3 | Methods detail (sampling + analysis for both) | ✓ Completed | Methodology 2.3, 2.6 |
| 4 | Integration (how QUAN + QUAL were combined) | ✓ Completed | Methodology 2.6.4, Results 3.5 |
| 5 | Method limitations (mixed-methods specific) | ✓ Completed | Discussion 5.2 (new paragraph) |
| 6 | Insights from integration (new knowledge) | ✓ Completed | Results 3.5, Discussion 4.1 |

**Table-3: Construct Validity and Reliability of Latent Variables: Indicators, Factor Loadings, and Model Fit**

| Latent Construct | Indicator Code | Indicator Description | Standardized Factor Loading | AVE (√AVE) | CR |
| --- | --- | --- | --- | --- | --- |
| **Policy Implementation Perceptions (PIP)** | PIP1 | Minors can easily purchase tobacco products | > 0.40* | 0.50 (0.71) | 0.84 |
|  | PIP2 | Tobacco industry influences policy implementation | > 0.40* |  |  |
|  | PIP3 | Health warnings are effective in discouraging smoking | > 0.40* |  |  |
|  | PIP4 | Smoke-free laws are enforced in public places | > 0.40* |  |  |
|  | PIP5 | Government is serious about tobacco control | > 0.40* |  |  |
|  | PIP6 | Tobacco advertising ban is effectively implemented | > 0.40* |  |  |
| **Tobacco Use Behaviors (TUB)** | TUB1 | Current use status (binary: user/non-user) | > 0.40* | 0.48 (0.69) | 0.81 |
|  | TUB2 | Daily use frequency (continuous) | > 0.40* |  |  |
|  | TUB3 | Number of quit attempts (ordinal) | > 0.40* |  |  |
|  | TUB4 | Age of initiation (continuous, reverse-coded) | > 0.40* |  |  |
|  | TUB5 | Intention to quit (ordinal) | > 0.40* |  |  |
| **Health Outcomes (HO)** | HO1 | Chronic cough (self-reported, binary) | > 0.40* | 0.42 (0.65) | 0.79 |
|  | HO2 | Shortness of breath (self-reported, binary) | > 0.40* |  |  |
|  | HO3 | Oral health problems (self-reported, binary) | > 0.40* |  |  |
|  | HO4 | Chest pain/discomfort (self-reported, binary) | > 0.40* |  |  |
|  | HO5 | Frequent respiratory infections (self-reported, binary) | > 0.40* |  |  |

*Note.* AVE = Average Variance Extracted; CR = Composite Reliability; √AVE = square root of AVE (used for discriminant validity).

- p < 0.01 for all factor loadings based on confirmatory factor analysis (CFA).

**Supplementary Fit Statistics (Referenced in text):**

- **Discriminant Validity (Fornell-Larcker Criterion):** √AVE for each construct (PIP = 0.71; TUB = 0.69; HO = 0.65) exceeded the inter-construct correlations, confirming discriminant validity.
- **Model Comparison:** The three-factor model (PIP, TUB, HO) fit significantly better than a one-factor model (Δχ² = 124.7, Δdf = 3, *p* < 0.001).
